# Monitoring the efficiency of reversal on anti-Xa direct oral anticoagulants using point-of-care viscoelastic testing

**DOI:** 10.1186/s12959-024-00659-8

**Published:** 2024-10-08

**Authors:** Lars Heubner, Oliver Grottke, Oliver Vicent, Peter Markus Spieth, Jan Beyer-Westendorf

**Affiliations:** 1 Department of Anesthesiology and Intensive Care Medicine, Faculty of Medicine and University Hospital Carl Gustav Carus, TUD Dresden University of Technology, Dresden, Germany; 2 Department of Anesthesiology, University Hospital Aachen, RWTH, Aachen University, Aachen, Germany; 3 Division of Hematology and Hemostasis, Department of Medicine I, Thrombosis Research, Faculty of Medicine and University Hospital Carl Gustav Carus, TUD Dresden University of Technology, Dresden, Germany

**Keywords:** Viscoeleastic testing, Bleeding, Reversal, RVV-test, Andexanet alfa, Point-of-care, POC, VET, DOAC

## Abstract

Bleeding events in patients receiving direct oral anticoagulation (DOAC) can be life-threatening even at therapeutic DOAC plasma concentrations, as anticoagulation impairs hemostasis and should therefore be identified immediately after hospital admission. The anticoagulatory effects of DOAC are typically not measurable in standard coagulation tests, such as PT or aPTT. Specific calibrated anti-FXa-tests allow specific drug monitoring, but they are too time-consuming for critical bleeding events and are commonly not available for 24 h/7 days in routine care. However, recent advances in point-of-care (POC) viscoelastic testing (VET) have shown a promising approach for rapid and quantitative detection of DOAC plasma concentrations using the Russell viper venom factor V activator (RVV for FXa-inhibitors) test or the ecarin clotting time (thrombin inhibitors). In acute bleeding situations, direct FXa inhibitors can be reversed by specific antidote andexanet alfa or hemostasis can be improved by prothrombin complex factor concentrates (PCCs). After reversal, confirmation of reversal efficacy is often requested, but no routine assays are currently available. Thus, the emergency management of bleeding DOAC patients is usually “blinded” with regard to reversal efficacy. POC VET laboratory assays might therefore also be helpful for measuring DOAC effects after reversal. We present a case series demonstrating the usefulness of RVV-clotting time post-DOAC reversal with andexanet alfa.

## Introduction

Direct oral anticoagulants (DOACs), including anti-FXa drugs (rivaroxaban, apixaban and edoxaban) and the anti-FIIa drug dabigatran (a thrombin inhibitor), are widely used for the prevention and treatment of thromboembolic events [1–4]. In general, DOACs are safer than vitamin K antagonists, but with the increasing number of DOAC prescriptions, patients with DOAC-related bleeding events are frequently encountered in emergency care [5–8] and can be life-threatening even at therapeutic DOAC plasma concentrations, as anticoagulation impairs hemostasis and should therefore be identified immediately after hospital admission. At the same time, accidental or intentional overdosing of DOACs sometimes occurs, as does chronic DOAC overexposure in patients with a decline in renal function or with drug interactions [9, 10], indicating a need for DOAC quantification to guide emergency management decisions. Specific antidots (andexanet alfa [11] or idarucizumab [12]) or nonspecific hemostatic treatment options, including prothrombin complex concentrates (PCCs) [13], are available to counteract the anticoagulant effects of DOACs. However, these interventions are expensive and carry a risk of thrombosis. The ANNEXA-I study showed that the use of andexanet alfa was associated with greater hemostatic efficacy than usual care but also a greater rate of thromboembolic events [14]. Interestingly, 33% of all patients in the andexanet alfa group and 46.9% in the usual care group failed to reach sufficient hemostatic efficacy. These findings highlight the need for further research and a patient-tailored approach. The fast and reliable quantification of DOAC plasma levels is especially important in emergencies to inform decision-making for or against DOAC reversal.

The anticoagulatory effects of DOAC at clinically relevant levels are typically not measurable in standard coagulation tests, such as PT or aPTT. Specific calibrated anti-FXa-tests allow specific drug monitoring, but they are too time-consuming for critical bleeding events and are commonly not available for 24 h/7 days in routine care.

Recent developments in point-of-care (POC) viscoelastic testing (VET) using specific test assays (the Russel venom viper test and the ecarin test) [15] have demonstrated the reliability of detecting relevant DOAC plasma concentrations to support rapid decision making in emergency situations [16–18].

After DOAC reversal, confirmation of reversal efficacy may be clinically warranted in cases of rebleeding or DOAC concentration rebound, but no routine assays are currently available. Unspecific reversal with PCC will not affect anti-Xa activity or DOAC concentration measurements. The effects of andexanet alfa cannot be measured with standard anti-Xa activity assays [19, 20] because the standard dilution protocols for anti-Xa chromogenic tests (e.g., STAGO dilution 1:44) lead to ex vivo dissociation of andexanet alfa and factor-FXa inhibitor molecules and, consequently, to an overestimation of anti-FXa levels. Theoretically, andexanet alfa efficacy can be measured with a modified anti-FXa test, but these assays are currently unavailable in routine care. Thus, the emergency management of bleeding DOAC patients is usually “blinded” with regard to reversal efficacy. The standard available calibrated anti-FXa measurements fail to indicate the reversal effect of andexant alfa in the case of anti-FXa DOACs. Here, we present a case series of DOAC reversals involving pre- and postreversal DOAC monitoring with POC VET.

## Methods

Standard laboratory analyses were performed as part of the clinical routine and included PT, aPTT, fibrinogen, fibrin monomers and D-dimer. Analyses were performed on STA R Max3-Analyzers (STAGO Deutschland GmbH, Düsseldorf, Germany), and the concentration of direct anti-FXa inhibitors was inferred from the one-stage chromogenic assay STA^®^-Liquid Anti-Xa assay (Stago Deutschland GmbH, Düsseldorf, Germany) on an STA R Max3-Analyzer (STAGO Deutschland GmbH, Duesseldorf, Germany). Blood samples for both conventional laboratory and POC testing were drawn from each patient at baseline (i.e. prior to reversal therapy) and at different time points after PCCs or the bolus of andexanet alfa as well as during/after the infusion of andexanet alfa and several hours after the reversal therapy was completed.

ClotPro^®^ Version 1.45a – serial number 2347 (Haemonetics, Boston, Massachusetts, United States) is a recently developed VET system that uses a cup and a pin to measure clot formation, with the cup rotating via an elastic element and the pin functioning as a stationary counterpart [21]. All measurements were performed following the manufacturer’s guidelines using a test-specific syringe for pipetting 340 µL of citrated patient blood per test and releasing it into the cups. The samples were processed for 60 min under standardized operating conditions at 37 °C within a maximum of two hours after arterial blood sampling. The recent addition of the snake venom RVV-V (Russell viper venom factor V activator) or ECA (Ecarin clotting time) assay provides the possibility for rapid detection of FXa inhibitors as well as low molecular weight heparin (RVV-V) and IIa inhibitors (ECA) [22, 23].

The clotting time (CT) was defined as the time from the start of the test until a clot amplitude of 2 mm was reached. The RVV assay involves a factor X activator derived from the snake venom “Russell’s viper”. The presence of factor Xa inhibitors prolongs coagulation time. The reference range for nonaccelerated CT in the RVV assay is < 80 s. As a functional test, the RVV test does not discriminate between low molecular weight heparin inhibition of Factor Xa or factor Xa inhibitor effects. The remaining blood from the laboratory analyses was centrifuged and frozen at -80 °C for further measurements. The Coamatic^®^ Heparin chromogenic assay (Diapharma, USA) was used to measure specific nondiluted anti-Xa values after andexanet alfa reversal at the university hospital in Aachen using a protocol previously described by Lu et al. [24, 25]. Written consent for publishing anonymized case details and images was obtained from all patients.

## Results

### Patient 1 (male, 78 years)

**Fig. 1:**
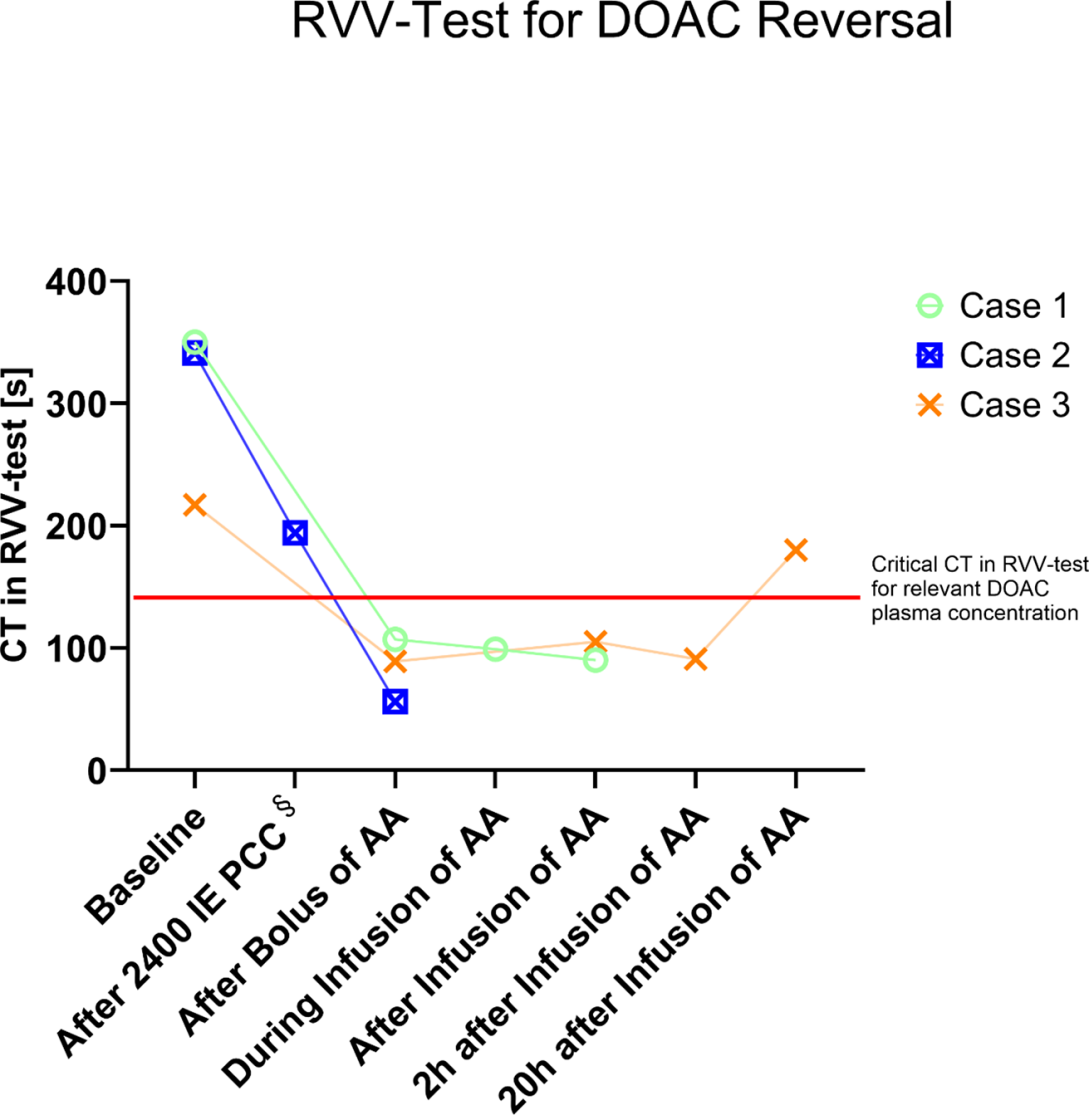
Clotting time (CT) in the RVV test measured by point-of-care viscoelastic testing comparing relevant time points (pre- and postreversal) of all three patients. The red line indicates a CT of 150 s, which seems to be a critical value for a DOAC plasma concentration with a relevant contribution to bleeding tendency AA: Andexanet alfa, CT: clotting time, RVV-test: Russell-Venom-Viper test, DOAC: direct oral anticoagulant,, PCC: prothrombin complex concentrate § only for case 2

was admitted to the emergency department (ED) and was suspected of having cerebral stroke with paraplegia of both arms and aphasia after an unobserved fall domestically. Whole-body computed tomography revealed dislocation fracture of the cervical spine with subsequent spinal cord injury and spinal hematoma. The pharyngeal airway was compressed by a prevertebral hematoma. Her medical history suggested the intake of an anti-FXa inhibitor, but type, dosage and last intake were unknown on admission. The clotting time (CT) in the POC VET RVV test, immediately performed at admission, demonstrated a prolongation to 350 s (reference range 48–77 s), and with a delay of 45 min, emergency laboratory analysis revealed a calibrated rivaroxaban concentration of 223 ng/ml (Table 1), correlating to therapeutic intensity anticoagulation. In line with the Summary of Product Characteristics (SmPC), high-dose andexanet alfa was administered (800 mg bolus over 30 min, followed by an infusion of 960 mg over 2 h), and the patient was transferred to the operating room (OR). Repeated POC VET measurements revealed a reduction of CT in the RVV test to 107 s after bolus, to 99 s after 1 h of infusion and to 90 s after the whole infusion of andexanet alfa was administered to the patient over 2 h (Fig. 1). During spinal surgery, hemostasis was clinically judged as “normal”, no further hemostatic treatments were necessary, and no further bleeding events were observed during the hospital stay. The patient was discharged 5 days after surgery in stable condition to the neurological rehabilitation clinic. During the short hospital stay, no thromboembolic events were observed.

**Table 1:** Laboratory parameters at baseline for all three cases. This table presents the values of the central laboratory of the clinic and the RVV test measured with point-of-care viscoelastic testing at baseline. PT: prothrombin time, aPTT: activated partial thromboplastin time, CT: clotting time, RVV-test: russell-venom-viper test

Baseline values	Case 1	Case 2	Case 3
PT [%]	31	38	55
aPTT [s]	36	35	27
Fibrinogen [g/L]	3.94	2.86	2.71
anti-Xa [ng/ml]	223	438	311
CT RVV [s]	350	341	217

### Patient 2 (female, 77 years)

presented at the ED with paraplegia of both legs and incontinence following severe back pain. The medication included rivaroxaban 20 mg OD. Magnetic resonance imaging (MRT) revealed severe intervertebral disc prolapse with the need for urgent surgery. On admission to the ED, the CT in the RVV test was prolonged to 341 s (reference range 48–77 s), and specific anti-FXa measurements later confirmed a supratherapeutic rivaroxaban plasma concentration of 438 ng/ml (Table 1). Prior to emergency surgery, the patient received a bolus of 2400 units of 4-factor PCC (correlating to a weight-adjusted dosage of 30 IU/kg). Due to uncontrollable epidural bleeding during surgery, repeated POC VET was performed. Although normal values were obtained for maximum clot firmness in the extrinsic, intrinsic and fibrinogen assays and CT was only slightly prolonged in the extrinsic test assay (95 s, normal range < 65 s), the DOAC-specific RVV test CT was still considerably prolonged at 194 s. Based on the clinically relevant bleeding and the discrepancy between standard CT and RVV CT, we considered a significant contribution of rivaroxaban activity even after PCC use. This led to the off-label administration of low-dose andexanet alfa (400 mg bolus over 15 min, 480 mg infusion over 2 h), which ultimately allowed successful surgery, preventing further epidural hematoma and cord damage. Following the andexanet alfa, the RVV-CT was fully corrected to within the normal range. (see Fig. 1). This case indicates the potential of RVV-CT-VET to detect insufficient DOAC reversal from the PCC at the bedside or during surgery and to confirm the hemostatic efficacy of andexanet alfa rescue therapy.

### Patient 3 (female, 88 years)

presented after severe trauma caused by a deep fall in a nursing home staircase. CT scans confirmed critical traumatic intracerebral and subarachnoid bleeding with multiple (open) craniofacial fractures, rib fractures and lung contusions with pleural effusion. Medical history indicated a presumed intake of apixaban 5 mg BID, with the last intake unknown. Routine assays in the POC VET at the ED could exclude trauma-induced coagulopathy, but CT in the RVV test was significantly prolonged to 217 s (reference range 48–77 s; see Fig. 1; Tables 1 and 2), leading to immediate DOAC reversal with a high dose of andexanet alfa (800 mg bolus; 960 mg infusion), and delayed results from apixaban-calibrated chromogenic anti-Xa-assay measurements confirmed a therapeutic apixaban plasma concentration of 311 ng/ml. Repeated RVV-CT tests revealed immediate reversal efficacy (89 s after bolus, 91 s after infusion) and moderate apixaban rebound 20 h after baseline (RVV-CT 180 s).

**Table 2:** Laboratory parameters over time (pre- and postreversals) for patient 3. This table presents the values of the central laboratory of the clinic, the RVV test measured with point-of-care viscoelastic testing and the post hoc measured values in the experimental laboratory. PT: prothrombin time, aPTT: activated partial thromboplastin time, GFR: glomerular filtration rate, CT: clotting time, RVV-test: russell-venom-viper test

		Emergency room (baseline)	After Bolus	After Infusion	2 h after end of Infusion	20 h after baseline
PT	%	55				59
aPTT	s	27				30
Fibrinogen	g/L	2.71				2.68
GFR	ml/min/1.73m^2^	66				75
Anti-Xa	ng/ml	311				
**CT RVV**	**s**	**217**	**89**	**105**	**91**	**180**
**Coamatic Heparin chromogenic assay**	**ng/ml**	**270**	**17**	**14**	**10**	**84**

Post hoc analysis of remnant blood samples using a nondiluted anti-Xa ELISA confirmed the correlation of these postreversal RVV-CT results for all time points: initially, a significant decrease in apixaban anti-Xa activity after AA bolus application remained low until 2 h after infusion, and subsequent apixaban rebounded 20 h after baseline (Table 2).

## Brief discussion

To date, evidence for measuring DOAC concentrations (or activity) following specific or nonspecific DOAC reversal is scarce, which is likely driven by a lack of routine tests to evaluate reversal efficacy. At the same time, phase III trials and observational studies have reported relevant proportions of patients in whom the clinical efficacy of DOAC reversal was not achieved or was complicated by DOAC rebound [14, 26–28]. Therefore, the validation of postreversal assays is an urgent unmet need in the emergency care of DOAC patients. The advantages of POC-VET include wide distributions in trauma centers, ease of use, and rapid whole-blood testing at the bedside [29]. Our case series indicated that the development of RVV-CT assays for POC-VET further increases the utility and clinical impact of POC-VET, as it can reliably detect and quantify apixaban, rivaroxaban and edoxaban anticoagulation; can inform decision making for reversal strategies; can confirm the hemostatic efficacy of this reversal (or a lack of effect); and can identify late DOAC rebound at the bedside. However, even using a functional whole blood test with the RVV agents, it faces the same limitations as all ex vivo test assays as it is an artificial test and partially overruling the coagulation cascade by its strong FXa activation. Therefore, the clinical relevance and interpretation needs further investigations. Large validation studies are warranted to evaluate our single-center experience at a larger scale, but if confirmed, the utilization of RVV-CT in routine emergency care will streamline the care of DOAC patients globally, which hopefully will then translate into improved clinical outcomes.

## Data Availability

No datasets were generated or analysed during the current study.
